# Intra-tumoral lymphocyte scoring in colorectal cancer: improving prognostic utility and correlation with underlying cancer biology

**DOI:** 10.3389/fgstr.2024.1493949

**Published:** 2024-11-28

**Authors:** Patrick L. Wagner, Jianwu Xie, Devin C. Flaherty, Hyun Park, Chelsea Knotts, Justine Debelius, Curtis Tilves, Neda Dadgar, Kunhong Xiao, Ali H. Zaidi, Louis Gil Acevedo, William LaFramboise, Ni Zhao, Cynthia Sears, Noel Mueller

**Affiliations:** ^1^ Allegheny Health Network Cancer Institute, Pittsburgh, PA, United States; ^2^ Winchester Medical Center, Winchester, VA, United States; ^3^ Centre for Translational Microbiome Research (CTMR), Department of Microbiology, Tumor and Cell Biology (MTC), Karolinska Institute, Stockholm, Sweden; ^4^ Department of Epidemiology, Johns Hopkins University Bloomberg School of Public Health, Baltimore, MD, United States; ^5^ Translational Hematology and Oncology Research, Enterprise Cancer Institute, Cleveland Clinic, Cleveland, OH, United States; ^6^ Center for Proteomics and Artificial Intelligence, Center for Clinical Mass Spectrometry, Allegheny Health Network Cancer Institute, Pittsburgh, PA, United States

**Keywords:** colorectal cancer, intra-tumoral lymphocytes, CD3/CD8 quantification, prognostic biomarkers, immunoscore

## Abstract

**Background:**

Intra-tumoral lymphocytes hold prognostic and predictive significance in colorectal cancer (CRC). The internationally validated Immunoscore™ predicts CRC survival risk by averaging percentile scores of tumor-associated CD3^+^ and CD8^+^ cell densities, but is limited by increased cost, intra-tumoral heterogeneity and omission of other immunologic variables of importance. To address these limitations, we sought to explore alternative prognostic markers based on CD3^+^ and CD8^+^ quantification in CRC.

**Methods:**

201 resected CRCs were subjected to quantitative CD3/CD8 immunohistochemistry, from which percentile cell counts were averaged (“I-score”) in a manner analogous to the Immunoscore™. I-score and exploratory endpoints, including CD3^+^ and CD8^+^ cell densities/percentiles, CD3^+^-CD8^+^ density/percentile differences, and CD3^+^:CD8^+^ density/percentile ratios were tested for association with clinicopathologic and genomic correlates and disease-specific survival (DSS).

**Results:**

CD3^+^ density among CRCs was right-skewed and potentially bimodal, while CD8^+^ density was right-skewed. Density and intra-tumoral variability for CD3^+^ and CD8^+^, as well as combination metrics including I-score, CD3^+^-CD8^+^ density/percentile differences, and CD3^+^-CD8^+^ density/percentile ratios showed distinct clinicopathologic and genomic associations, suggesting that each may hold unique biological significance. CD3^+^ density, CD8^+^ density/percentile, I-score and CD3^+^:CD8^+^ percentile ratio were associated with DSS; only CD3^+^:CD8^+^ percentile ratio was pTNM stage-independent on multivariable analysis. Independently, CD8^+^ density was as prognostic as I-score, questioning the necessity of CD3, or a combination metric.

**Conclusions:**

I-score, in our study, was closely associated with potentially confounding biologic variables such as sex, active smoking, pTNM stage, and mutations in BRAF, and MMR genes. More precise and biologically relevant biomarkers can be achieved by using data-driven CD3^+^/CD8^+^ density cutoffs and ratios, while controlling for important clinicopathologic and molecular variables in CRC. Independent validation and inclusion of other relevant immunocyte types could bring these findings closer to clinical utility in CRC.

## Introduction

Colorectal cancer (CRC) remains a major global health burden, representing a significant cause of cancer-related morbidity and mortality (1). Advances in understanding the tumor immune microenvironment have paved the way for immunotherapeutic interventions in CRC and other cancers, but few patients with CRC are currently eligible for available agents like immune checkpoint inhibitors (ICIs) (2, 3). Intra-tumoral lymphocyte counts, particularly CD3^+^ and CD8^+^ cells, have emerged as important prognostic biomarkers by providing insight into disease progression and response to immunotherapy (4, 5). Immunoscore™, a well-established immune-based prognostic tool, has significantly contributed to risk stratification and treatment decision-making in CRC patients (6). This scoring system relies on averaged percentile values of CD3^+^ and CD8^+^ cell densities within the tumor tissue to predict patient outcomes.

Despite its validated association with poor prognosis, the Immunoscore™ has important limitations. First, its utility as an independent biomarker is limited by the lack of a comprehensive assessment of potential confounding variables, including clinicopathologic factors or tumor genomic profiling (7). Second, the arbitrary conversion of lymphocyte counts into percentile bins might lead to potential distortions in the analysis of non-normally distributed underlying biologic variables (8). Third, the Immunoscore™ might be subject to sampling bias, for example, in cases of significant intra-tumoral heterogeneity (9, 10). Fourth, the commercialization of an assay that is ultimately based on routine immunostaining methods (CD3 and CD8) and simple density counting may introduce unnecessary costs and delays in patient care. Fifth, by focusing exclusively on CD3^+^ and CD8^+^ cells, the Immunoscore™ may omit other immune cell populations of importance (7). Finally, because specific biomarkers relevant to checkpoint inhibition or lymphocyte exhaustion are not included in the Immunoscore™, it has gained little traction in predicting eligibility for or response to available immunotherapeutic agents, such as ICIs (11, 12).

To address some of these limitations of the Immunoscore™ and other biomarkers of tumor lymphocyte infiltration, the present research revisits the assessment of CD3^+^ and CD8^+^ cell density quantification in CRC, incorporating clinical, pathological, and genomic factors, as well as different individual and combination metrics reflecting lymphocyte counts. We hypothesized that CD3^+^ and CD8^+^, when assessed individually as independent variables, would be associated with overlapping, but distinct, clinical and pathologic factors. It is hoped that by refining immune profiling of CRCs, new biomarkers will emerge with enhanced applicability in clinical practice, ultimately guiding personalized treatment decisions and improving patient outcomes in the context of immunotherapy for colorectal cancer.

## Methods

### Patient cohort selection and tissue samples

A retrospective cohort of 201 CRC patients who underwent surgical resection of their primary colorectal tumors between 2016 and 2019 at Valley Health Winchester Medical Center (Winchester, VA, USA) was selected for analysis. Consecutive cases with adequate tissue availability were included. The facility’s catchment area has a predominantly white and rural population base. Due to the unpredictable impact of chemotherapy or radiation on the tumor immune microenvironment, patients with prior chemotherapy or radiation were excluded.

Formalin-fixed, paraffin-embedded (FFPE) tumor tissue samples were selected from each patient and reviewed to confirm the diagnosis and ensure adequate tumor representation. The best full cross-sectional histology block, including central and peripheral aspects of the tumor, was selected from each case after review by two pathologists (PLW and JX) for tissue sectioning and tumor retrieval. Clinical and demographic data, including age, sex, tumor location, American Joint Committee on Cancer pathologic tumor/node/metastasis (pTNM) stage, and survival outcomes, were obtained from medical records and pathology reports. The study was approved by the Winchester Medical Center Institutional Review Board (Protocol # 2020-1201) and waiver of consent was granted for this expedited-review research type (2018 Common Rule Category 5).

### Quantitative CD3/CD8^+^ immunohistochemistry

FFPE tissue from each tumor, with immediately adjacent non-neoplastic colon tissue, was verified to contain malignant tissue independently by two pathologists. Immunohistochemistry (IHC) was performed on 4 µm-sections with CD3 (PA0553, Leica Biosystems, Deer Park, IL, USA) and CD8 (PA0183, Leica Biosystems) primary antibodies using the BOND RXm (Leica Biosystems). Antigen retrieval was performed with ER-2 solution (Leica Biosystems) for 20 minutes and the detection step was completed with BOND Polymer Refine Detection kit (DS9800, Leica Biosystems). Mounted slides were then scanned using an Aperio Versa 8 scanner (v 1.0.4.125, Leica Biosystems) at 10X.

Image analysis was then performed after import into quantitative image analysis software (ImageJ, https://imagej.net/ij/index.html). A cell counting algorithm was manually tuned to optimal discrimination of DAB (3,3′-Diaminobenzidine)-stained cells. On each slide, the entire available area of malignant epithelium, along with immediately adjacent (within 0.5mm) stromal tissue was then used for cell count analysis, to maximize the total area analyzed in each case. Non-neoplastic adjacent tissue was excluded from analysis. Cell counts and the amount of cross-sectional area examined were then recorded and used to calculate cell densities. In order to assess intra-tumoral variation in cell densities, the covariance of cell density was utilized by arbitrarily dividing the region of interest in each case into three separate units; cell densities were calculated and recorded separately for each area and covariance was calculated. The mean and median total examined area per case were 37.3 and 27.4 mm^2^, respectively.

### Calculation of I-score and novel metrics

The percentile scores for CD3^+^ and CD8^+^ cell densities were calculated for each patient, and the “I-score,” analogous to the Immunoscore™, was generated by averaging the CD3^+^ and CD8^+^ percentile scores in each case. Central and peripheral aspects of each tumor, including immediately peri-tumoral stromal tissue within 0.5mm of malignant cells, were included in the analysis, but not scored separately. Novel metrics, including CD3^+^-CD8^+^ density/percentile differences and CD3^+^:CD8^+^ density/percentile ratios, were computed to explore candidate biomarkers within the patient cohort.

### Next-generation sequencing

DNA extraction from tumor samples was performed using the High Pure FFPE DNA Isolation Kit (Roche Diagnostics, Indianapolis, IN, USA). Whole-exome next-generation sequencing (NGS) results (GENEWIZ™, Azenta Life Sciences, NJ, USA) using the Illumina platform were employed to assign mutation status for selected genes of interest in colorectal cancer. tp53 and KRAS genes were analyzed due to their high prevalence and established roles in colorectal carcinogenesis, whereas BRAF and mismatch repair (MMR) gene mutations were analyzed due to their known association with lymphocyte density in CRC. MMR genes were analyzed as a collective group, since their individual prevalence was too low to achieve adequate statistical power for subgroup analysis in this cohort. Single nucleotide variations were considered to be clinically significant mutations when classified as “deleterious” using Sift (https://sift.bii.a-star.edu.sg/) or as “possibly damaging”/”probably damaging” by Polyphen (http://genetics.bwh.harvard.edu/pph2/).

### Statistical analysis

Clinicopathologic and genomic variables were recorded from each case, including age, biologic sex, smoking status, alcohol use, body mass index (BMI), tumor side (right *vs*. left colon), family CRC history (first-degree relative), chronic kidney disease, type 2 diabetes, carcinoembryonic antigen (CEA) level, tumor size, tumor grade, lymphovascular invasion, pTNM stage, or mutation in BRAF, KRAS, TP53, or any mismatch repair (MMR) gene, and tumor mutation burden. These variables were analyzed for association with CD3^+^ and CD8^+^ densities, I-score, and exploratory metrics using non-parametric tests of association. For categorical variables, the Mann-Whitney (two groups) and Kruskal-Wallis (three or more groups) tests were utilized. For continuous variables, Spearman’s rank correlation method was used. For continuous variables, tests of normality based on skewness and kurtosis were carried out using the Stata *sktest* function (https://www.stata.com/manuals/rsktest.pdf).

Disease-specific survival (DSS) was defined as the interval from initial diagnosis to death from CRC obtained from review of the electronic medical record. Patients were censored when they were lost to follow-up or died of other causes, with a total follow-up time for the entire cohort of 605 person-years. Cox proportional hazards models were used to test association of DSS with CD3^+^ and CD8^+^ densities, I-score, and novel metrics as continuous variables. To assess whether lymphocyte density contributed prognostic value beyond conventional AJCC pathologic (pTNM) staging, we-assessed stage-independence using multivariable analysis in which pT, pN and pM stage strata were added individually as covariates.

Stata (version 16.1, StataCorp, College Station, TX, USA) was used to perform tests of association and Cox proportional hazards modeling. Statistical significance was defined as p value <0.05. Multiple comparisons were addressed by controlling the Benjamini-Hochberg false discovery rate at 0.05 level.

Optimization of cell count and combination metric cutoffs for prognostic stratification was performed using an open-access web application (Cutoff Finder, https://molpathoheidelberg.shinyapps.io/CutoffFinder_v1/), as previously described (13). Briefly, the application fits candidate Cox proportional hazard models for a time-to-event outcome by dichotomizing at every observed value of an independent continuous variable, using the *coxph* and *survfit* functions from the R package *Survival* (http://cran.r-project.org/package=survival). The dichotomization point with the smallest p value in survival outcome by log-rank test is defined as the optimal “cutoff”.

## Results

### Study population

The clinical and demographic characteristics of the patients are presented in **Table 1**. To assess potential bias introduced by exclusion of pretreated patients and those not undergoing surgery, baseline patient and tumor characteristics were compared with 282 patients that were treated in the same health system for CRC during the same years but were not included in this retrospective cohort. As expected due to these exclusion criteria, included patients were less likely to have rectal cancer (12.4% vs. 34.8%); therefore, right-sided tumors—defined as cecum/ascending colon/hepatic flexure—were disproportionately higher in the included group (42.4% vs. 27.4%, p<0.001). Included participants also had tumors with lower proportions of grade 1, pT1, pM1 and overall pTNM stage IV (p=0.002). The remaining underlying patient demographic and clinical variables did not vary between included and excluded patients (**Supplementary Table S1**).

**Table 1:** Clinical and pathologic characteristics of 201 tumors analyzed in this study.

Patient or Tumor Characteristic		Frequency (%)	Mean +/- SD	Range
Age at diagnosis (years)			67.5 +/- 13.4	21, 95
Early onset (<50 years) CRC	No	182 (90.6)		
	Yes	19 (9.5)		
Sex	Female	94 (46.8)		
	Male	107 (53.2)		
Body mass index (kg/m^2^)			29.5 +/- 6.8	15.7, 60.0
First-degree relative with CRC	No	154 (87)		
	Yes	23 (13)		
Active smoking	No	178 (88.6)		
	Yes	23 (11.4)		
Pack-years			11.0 +/- 17.4	0, 75
Alcohol use	No	157 (78.5)		
	Yes	43 (21.5)		
Chronic kidney disease	No	172 (85.6)		
	Yes	29 (14.4)		
Type II diabetes	No	150 (74.6)		
	Yes	51 (25.4)		
Tumor site	Left colon	94 (47)		
	Right colon	106 (53)		
Tumor size (cm)			4.9 +/- 2.4	0.8, 15
Carcinoembryonic antigen level (ng/mL)			72.2 +/- 526.4	0.5, 6570
pT stage	pT1	8 (4.0)		
	pT2	31 (15.6)		
	pT3	117 (58.8)		
	pT4	43 (21.6)		
pN stage	pN0	99 (49.5)		
	pN1	69 (34.5)		
	pN2	32 (16)		
pM stage	pM0	171 (85.1)		
	pM1	30 (14.9)		
Overall pTNM stage	I	32 (15.9)		
	II	66 (32.8)		
	III	75 (37.3)		
	IV	28 (13.9)		
Lymphovascular invasion	No	106 (63.9)		
	Yes	60 (36.1)		

SD, standard deviation; CRC, colorectal cancer.

### CD3^+^ and CD8^+^ cell density and covariance

**Figure 1:**
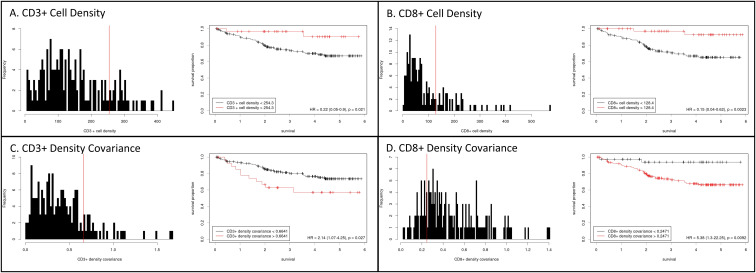
Histograms of cell density for CD3+ **(A)** and CD8+ **(B)** cells within colorectal cancers. CD3+ cell density displayed a right-skewed and potentially bimodal distribution, while CD8+ cell density was right-skewed and kurtotic. Kaplan-Meier curves for disease-specific survival were generated after separating cases at the indicated optimal cutpoints (Cutoff Finder, https://molpathoheidelberg.shinyapps.io/CutoffFinder_v1/; see methods). Intra-tumoral heterogeneity, assessed as covariance, for CD3+ **(C)** and CD8+ **(D)** cell density, with survival analysis stratified at the indicated cutpoints.

CD3^+^ cell density exhibited a right-skewed and potentially bimodal distribution, suggesting that distinct subpopulations of tumors may exist with varying levels of CD3^+^ cell infiltration (p=0.0001, **Figure 1**). In contrast, CD8^+^ cell density displayed kurtosis (p<0.0001), with a right-skewed (p<0.0001) distribution (**Figure 1**). In addition to evaluating the cell density distributions, we also assessed covariance as a surrogate metric of intra-tumoral heterogeneity for CD3^+^ and CD8^+^ cell density (**Supplementary Figure S1**). For both CD3^+^ and CD8^+^ cells, covariance was right-skewed (p<0.0001) and inversely proportional to cell density (p<0.0001); CD3^+^ density covariance also exhibited kurtosis (p<0.0001).

### Associations of clinical, pathologic and genomic factors with CD3^+^ and CD8^+^ cell density and covariance

We separately assessed CD3^+^ and CD8^+^ cell density for association with clinical and pathologic factors. CD3^+^ cell density was associated with female sex (p=0.03), non-active smoking status (p=0.02), and earlier pTNM stage (p ≤ 0.01). CD8^+^ cell density was associated with advanced age (p=0.006), non-active smoking status (p=0.02) and earlier pTNM stage (p<0.001; **Table 2**; **Supplementary Figure S2**). CD3+ or CD8+ cell density was not associated with alcohol use, body mass index (BMI), tumor laterality (right *vs*. left colon), family history, diabetes, nor chronic kidney disease.

**Table 2:** Association of clinical, pathologic and mutational features with CD3+ density, CD8+ cell density and I-score.

		CD3+ cell density			CDB+ cell density			I-score		
	n	ρ	mean [95%CI]	p	ρ	mean [95%CI]	p	ρ	mean [95%CI]	p
Age		0.12		0.1	0.2		0.006*	0.17		0.02
Sex										
Female	94		167.9 [145.8,190.1]	0.03		108.6 [85.4,131.9]	0.05		55.0 [49.1,60.8]	0.04
Male	107		134.1 152.2			74.4 [61.4,87.5]			46.2 [40.9,51.6]	
Body mass index		-0.03		0.7	-0.06		0.4	-0.03		0.7
First degree relative										
No	154		152.9 [135.7,170.0]	0.5		89.9 [74.4,105.5]	0.3		50.1 [45.4,54.8]	0.7
Yes	23		134.7 [92.9,176.6]			111.1 [67.7,154.5]			52.5 [38.5,66.4]	
Active smoker										
No	178		156.5 [141.0,172.0]	0.02		94.8 [80.3,109.2]	0.04		52.2 [48.0,56.5]	0.02
Yes	23		106.6 [73.8, 139.4]			59.8 [35.7,83.9]			37.9 [27.3,48.6]	
Alcohol use										
No	157		153.0 [135.8,170.2]	0.8		96.9 [80.9,112.9]	0.3		51.2 [46.4,55.9]	0.5
Yes	43		138.7 [114.3, 163]			67.3 [50.6,84.0]			47.3 [40.4,54.3]	
Right-sided tumor										
No	94		150.4 [131.1, 169.8	0.8		86.9 [69.9,103.9]	0.9		50.8 [45.4,56.2]	1.0
Yes	106		149.7 [128.2,171.2]			94.5 [74.5, 114.5]			50.2 [44.2,56.1]	
Lymphovascular invasion										
No	106		167.8 [148.3, 187.3	0.02		104.9 [85.0,124.9]	0.02		56.3 [51.1,61.6]	0.01
Yes	60		131.0 [105.1, 156.9]			75.5 [55.3,95.7]			44.6 [37.2,51.9]	
Grade										
1	10		137.2 [107.3, 167.2	1.0		78.4 [49.0,107.7]	0.8		53.0[41.4,64.5]	0.9
2	151		149.2 [133.8, 164.6			87.3 [73.4,101.2]			51.1 [46.8,55.4]	
3	34		165.9 [118.0, 213.9			110.9 [65.9, 155.9]			48.8 [36,61.6]	
T stage										
1	8		250.0 [169.3, 330.8	0.001**		115.6 [30.3, 200.9]	<0.001**		70.7 [53.7,87.7]	<0.001**
2	31		150.7 [115.9, 185.6			128.8 [90.0, 167.5]			57.6 [48.4,66.9]	
3	117		163.2 [143.8, 182.7			95.6 [77.6,113.5]			53.3 [48.2,58.5]	
4	43		99.6 [78.2, 120.9]			51.2 [36.5, 65.8			35.1 [27.3,42.8]	
N stage										
0	99		174.5 152.9, 196	0.003*		115.4 [93.0,137.9]	0.001**		58.4 [52.8,64.0]	<0.001**
1	69		136.5 [113.2, 159.9]			71.9 [55.2,88.7]			45.2 [38.7,51.8]	
2	32		107.7 [81.1, 134.3			62.1 [39.4,84.8]			39.2 [30.1,48.2]	
M stage										
0	171		157.9 [141.9, 173.8	0.01		97.9 [83.2, 112.6	<0.001**		53.2 [49.0,57.5]	<0.001**
1	30		106.5 [80.8,132.2]			46.8 [28.2,65.5]			34.4 [25.4,43.5]	
BRAF mutation										
No	151		141.5 [126.2, 156.8	0.1		75.0 [63.4, 86.6]	<0.001**		47.1 [42.6,51.6]	0.01
Yes	50		175.5 [141.8, 209.1			138.0 [101.3, 174.7]			60.3 [52.4, 68.2	
KRAS mutation										
No	181		152.7 [137.4,168.0]	0.3		89.2 [75.3,103.2]	0.6		50.6 [46.5,54.8]	0.8
Yes	20		129.5 [88.1, 170.9			100.7 [60.4, 141.1]			48.6 [35.0,62.3]	
tp53 mutation										
No	153		146.8 [129.8, 163.8	0.3		94.0 07.77.110.3	0.9		49.8 [45.0,54.5]	0.5
Yes	48		159.6 133.1, 186.2			79.5 [60.9,98.1]			52.3 [45.2,59.5]	
MMR mutation										
No	149		139.6 [124.4,154.9]	0.04		75.3 [64.4,86.1]	0.01*		47.6 [43.1,52.0]	0.02
Yes	52		180.9 [147.6,214.2]			137.1 [98.1,176.2]			58.8 [50.7,67.0]	
Tumor mutational burden		-0.02		0.8	-0.14		0.06	-0.08		0.3

Unadjusted p value for comparison of continuous variables using Spearman’s rank correlation coefficient (ρ); unadjusted p value for categorical variables using Mann-Whitney test (two subgroups) or Kruskal-Wallis test (three or more subgroups). *, adjusted p value <0.05; **, adjusted p value <0.01 (Benjamini-Hochberg correction at FDR =0.05).

Selected genomic alterations were then assessed for association with CD3^+^ and CD8^+^ cell density (**Table 2**). CD3^+^ and CD8^+^ cell density were each significantly associated with the presence of deleterious mutations in any MMR gene, (p=0.04 and 0.01, respectively), while CD8^+^ density was also associated with mutation in BRAF (p<0.001).

Intra-tumoral variation in CD3^+^ cell density, as measured by CD3^+^ density covariance, was not associated with any of the clinicopathologic or genomic variables tested. CD8^+^ cell density covariance, however, was associated with tumor size (p=0.02), active smoking (p=0.05), and the presence of an MMR gene mutation (p<0.001).

### Combination and exploratory metrics: prognostic utility of established and exploratory metrics

The I-score, a combinatorial percentile-based metric calculated in a manner analogous to the Immunoscore™, was positively associated with age and female sex, and negatively associated with active smoking status (**Table 2**). I-score was negatively associated with key pathologic variables and staging metrics including pT stage, pN stage, pM stage, and lymphovascular invasion. I-score was associated with mutation in BRAF and any MMR gene.

We then generated additional exploratory combinations incorporating CD3^+^ and CD8^+^ counts. Theoretically, the subset of CD8^+^ cells, among all CD3^+^ lymphocytes, should contain cells capable of mounting an effective cytotoxic anti-tumor response. Conversely, CD3^+^CD8^-^ cells could be enriched with regulatory, suppressive or otherwise maladaptive cell types. We therefore hypothesized that measuring relative quantities of intra-tumoral CD3^+^ and CD8^+^ cells could identify biologically important tumor subgroups. This was assessed both in absolute (CD3^+^- CD8^+^ difference) and relative (CD3^+^:CD8^+^ ratio) terms, using both raw counts and percentile conversions.

**Figure 2:**
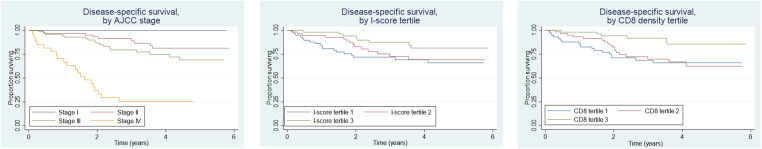
Disease-specific survival by AJCC stage, I-score tertiles, and CD8+ cell density tertiles in colorectal cancer. Higher AJCC stage correlates with poorer survival, while higher I-score and CD8+ density tertiles are associated with improved survival, highlighting the prognostic value of immune infiltration.

We next tested all the individual and combination metrics incorporating intra-tumoral CD3^+^ and CD8^+^ cells described above for association with DSS (**Table 3**). Univariable Cox proportional hazard regression analysis for DSS based on stage and I-score were initially performed for validation purposes and confirmed the expected prognostic significance of pTNM staging (p<0.001) and I-score (p=0.02, **Figure 2**). Additional clinical, pathologic, and genomic factors associated with poor survival were male sex, active smoking, alcohol use, CEA, tumor size and grade, and lymphovascular invasion (**Supplementary Table S3**). CD3^+^ and CD8^+^ cell densities were individually associated with favorable DSS, but the prognostic significance of CD3^+^ was lost when controlling for CD8^+^ count, suggesting that the true prognostic impact is based on the CD8^+^ subset. In fact, the use of CD8^+^ cell density alone generated prognostic groups essentially equivalent to the I-score, suggesting that the CD3^+^ count and combination metrics may add little additional value to simply counting CD8^+^ cells (**Figure 2**). Neither CD3^+^ nor CD8^+^ density was found to be independently predictive of DSS when added to a to a multivariable Cox model with pT, pN and pM as co-independent variables (**Table 3**).

**Table 3:** Cox proportional hazards models for disease-specific survival with established and exploratory metrics of CD3 and CD8 density.

	Univariable			Multivariable with pT, pN, pM stage		
	HR	95% CI	p	HR	95% CI	p
CD3 density	1.00	[0.992, 0.999]	0.03	1.00	[1.00, 1.00]	0.9
CD3 percentile	0.99	[0.98, 1.00]	0.05	1.00	[0.99, 1.01]	0.9
CD3 covariance	1.38	[0.55, 3.46]	0.5	1.56	[0.62, 3.93]	0.4
CD3 density >235/mm^2^	0.22	[0.05, 0.90]	0.02	0.84	[0.28, 2.52]	0.8
CD8 density	0.99	[0.986, 0.998]	0.01	1.00	[0.99, 1.00]	0.6
CD8 percentile	0.99	[0.976, 0.997]	0.01	1.00	[0.99, 1.02]	0.5
CD8 covariance	2.07	[0.79, 5.41]	0.1	0.68	[0.24, 1.95]	0.5
CD8 density >128/mm^2^	0.15	[0.04, 0.62]	0.009	0.46	[0.13, 1.58]	0.2
I-score	0.99	[0.974, 0.998]	0.02	1.00	[0.99, 1.02]	0.7
I-score tertile 2 (vs. 1)	0.78	[0.39, 1.56]	0.5	1.61	[0.69, 3.77]	0.3
I-score tertile 3 (vs. 1)	0.41	[0.18, 0.94]	0.04	1.15	[0.45, 2.96]	0.8
CD3-CD8 density difference	1.00	[1.00, 1.01]	0.5	1.00	[1.00, 1.01]	0.6
CD3-CD8 percentile difference	1.01	[0.99, 1.02]	0.3	1.00	[0.98, 1.01]	0.7
CD3:CD8 density ratio	1.07	[0.99, 1.15]	0.07	1.00	[0.90, 1.10]	0.9
CD3:CD8 percentile ratio	1.06	[1.03, 1.1]	<0.001	1.04	[1.01, 1.08]	0.02

HR, hazard ratio. 95%CI, confidence interval. Results for each metric are shown for univariate analysis. To assess stage-independence, each metric was also then in turn included in a multivariable regression model with pT, pN, and pM; for each model, the hazard ratio of the assessed metric in its multivariable regression with pT, pN and pM are shown.

Optimal cutoffs for DSS risk stratification were then determined for intra-tumoral CD3^+^ and CD8^+^ counts. A CD3^+^ count >254/mm^2^ (equivalent to the 83^rd^ percentile; HR = 0.22 [95%CI 0.05, 0.90], p=0.02) and a CD8^+^ cell density of >128/mm^2^ (equivalent to the 77^th^ percentile; HR = 0.15 [95%CI 0.04, 0.62], p=0.002) were each associated with favorable survival (**Figure 1**). Intra-tumoral heterogeneity of CD3 and CD8 cell density, as indicated by covariance, was also able to stratify survival risk at a covariance cutoff value of >0.66 for CD3^+^ (HR=2.14 [95% CI 1.07, 4.25], p=0.03) and >0.25 for CD8^+^ (HR=5.38 [95%CI 1.30, 22.30], p=0.009) () (**Supplementary Figure S1**).

Combinatorial metrics were also associated with DSS (**Table 3**). I-score, analogous to Immunoscore™, was significantly associated with improved survival (p=0.02) when assessed as a continuous variable, and an optimal percentile cutoff value of >46 was identified (HR=0.43, 95%CI [0.22, 0.81], p=0.007). The relative quantity of intra-tumoral CD3^+^ and CD8^+^ cells also appeared to show prognostic importance, with the CD3^+^:CD8^+^ percentile ratio and specifically a CD3^+^:CD8^+^ density percentile ratio cutoff of >0.62 being associated with DSS (HR=0.4, 95%CI [0.21, 0.76], p=0.004). The magnitude of the absolute difference between CD3^+^ and CD8^+^ percentile in a given case was also associated with shorter DSS at a cutoff of >15 percentile units (HR=2.1, 95%CI [1.10, 4.20], p=0.03). Among all combinatorial and exploratory metrics, only the CD3^+^:CD8^+^ percentile ratio was a significant *stage-independent* survival predictor when added to a multivariable Cox proportional hazards regression model incorporating pT, pN and pM stage, whereas I-score was not (**Table 3**).

## Discussion

Much effort has been focused over the past two decades in defining consensus molecular and immune subsets of CRC in order to define meaningful subgroups of patients for targeted therapy (14). In particular, the fundamental recognition of the cytotoxic (CD8^+^) T cell as the critical anti-tumor immune effector in CRC has led to the emergence of intra-tumoral lymphocyte density as a key prognostic biomarker in CRC (15–17). In this study, we found overlapping but distinct implications of CD3^+^ and CD8^+^ cell density counts within CRC, and identified absolute cutoffs for CD3^+^ and CD8^+^ that may define biologically meaningful prognostic subsets within CRC. We also found that the relative density of CD3^+^ and CD8^+^ cells (difference and ratio) harbors prognostic significance. In our study, CD3^+^ and CD8^+^ cell counts, along with combinatorial metrics including an analogous metric to the Immunoscore™, were closely associated with CRC stage, and when controlling for CRC stage these variables were not independently predictive of incident CRC mortality. Of all the investigated metrics based on CD3^+^ and CD8^+^ cell densities, only the CD8^+^:CD3^+^ percentile ratio was a stage-independent predictor of CRC mortality in this cohort.

Among the candidate biomarkers of immune contexture in CRC, the Immunoscore™ has gained the widest recognition in predicting patient outcomes (18). International validation of this stage-independent biomarker has been achieved across diverse populations and treatment settings, with large studies typically yielding a hazard ratio for DSS in the range of 0.2 for patients with high-Immunoscore™ tumors (19–21). Beyond its use as a global prognostic index, the Immunoscore™ has been assessed as a predictive tool in patient or regimen selection for adjuvant therapy in stage II/III CRC (22–24); as a risk-stratification metric in stage IV patients undergoing metastasectomy (25, 26); and as a predictor of recurrence in rectal cancer following a complete clinical response to neoadjuvant chemoradiation (27). In spite of the utility of the Immunoscore™ in these diverse clinical scenarios, the search for an optimal biomarker of immune contexture in CRC remains an active area of research (28–30), now more than 15 years after the seminal observations of Galon et al. (31) and a decade beyond the formation of international validating collaboratives (32).

Fundamentally, the Immunoscore™ is determined by generating density counts of CD3^+^ and CD8^+^ cells in the tumor center and tumor periphery (33). These raw counts are converted into percentiles, which are averaged in an unweighted fashion to generate a final percentile score (34). In most published works on this topic, the percentile scores are used to define arbitrary strata, such as tertiles or percentile cutoffs (e.g. <25, 25-70, >70) for use in survival analyses and generation of Kaplan-Meier curves (34). When non-normally distributed parameters are converted to percentiles, then sorted into arbitrary strata and combined, information loss is inevitable (8). It is possible that alternative methods, such as considering CD3^+^ and CD8^+^ individually as continuous variables, weighting them unequally, or analyzing relative cell counts and ratios instead of averaged counts, could add to the prognostic and predictive utility of intra-tumoral lymphocytes as biomarkers in CRC. To date, alternative strategies have not been well characterized.

By examining density distributions of CD3^+^ and CD8^+^ counts within tumors, we found putative absolute tumor cutoffs may represent biologically significant subsets of CRC that are blended or separated when converted to averaged percentile categories. CD8^+^ cell density alone within CRC can stratify patients into risk categories essentially indistinguishable from combined percentile scores, suggesting the inclusion of CD3^+^ and conversion to percentile groupings may be unnecessary. Although a number of ‘immune scores’ other than Immunoscore™ have been proposed, as reviewed by Malka et al., few have looked solely at CD8^+^ in isolation, with most also incorporating CD3^+^ and/or CD45R0^+^ cell counts (35). Semi-quantitative scales (36) and alternative, open-access software methods have been devised to validate alternative immune cell density scores that accomplish risk stratification at lower cost in wider practice settings (37). While a simpler assay may be more efficient and economically viable, quantification with data-driven cutoffs is necessary, as semi-quantitative impressions of immune cell density have been proven inferior to the Immunoscore™ (28, 37).

A single metric may not capture all the information. In the metastatic setting, Wang et al. illustrated that CD8^+^ and CD3^+^ cell densities, when considered independently, were associated with different predictive significance based on drug regimen. Because different immune cell types within CRC are likely to imply different biological meaning, additional effort should be devoted to studying the individual ramifications of immune cell subsets and their relative density within CRC. Staining for markers of T cell activation state, exhaustion, or of helper T cell subsets—such as T_H_1, T_H_2, T_H_17, T_REG_, or T_H_22 cells—could result in improved prognostic utility, while markers of checkpoint inhibition could be expanded (38). Integration of myeloid cell types has also been attempted in the form of a nomogram in which CD33^+^ myeloid-derived suppressor cell counts were added to CD8^+^ counts in predicting outcome in intermediate-stage CRC (39), and a macrophage score was proposed in the stage IV setting (26).

Incorporating other morphologic or molecular features into risk stratification biomarkers may further hone the predictive capacity of these tests. Tumor budding has been added to immune cell scoring in attempts to improve prognostic and predictive value, with the presence of these two factors and their spatial relationship being shown by machine learning to contribute prognostic information (40, 41). More sophisticated tests, based on high resolution spatial staining methods, transcriptomics, or liquid biopsies may ultimately replace IHC, but face barriers related to excessive relative cost. Emerging strategies to employ bulk transcriptomics to estimate CD3^+^ and CD8^+^ counts as an alternative to IHC staining has been successfully explored (26), but advanced molecular pathology techniques of this nature will require significant optimization, validation and cost reduction prior to gaining use in routine clinical practice. Clearly, as the breadth and variety of novel immunotherapeutic options expands in the coming years, biomarkers of immune contexture in CRC are expected to be in high demand (42), and publicly available datasets, such as the Cancer Genome Atlas (TCGA), may facilitate future studies of this type (43).

This study has limitations that require due consideration and follow-up analysis. The exploratory analysis included 201 tumors from a single US center, whereas internationally validated prognostic metrics typically contain thousands of patients from a variety of treatment settings. Although the I-score described here was carried out in a manner analogous to the Immunoscore™, our method considered entire cross-sections of tumors rather than separately analyzing and averaging lymphocyte counts from the tumor center and the invasive margin. This methodologic difference could account for disparate results relative to prior studies, and while a direct head-to-head comparison would be ideal in evaluating this possibility, it was not feasible in this retrospective dataset due to the proprietary and cost-prohibitive nature of Immunoscore™.

The overall external validity of our findings is supported by agreement with prior large cohort studies investigating the association of lymphocyte density with patient factors including smoking (44), BMI (45), BRAF and MMR mutations (36). Likewise, the association described here between intra-tumoral heterogeneity of lymphocyte density and prognosis in CRC is also in agreement with previous reports (46). Ultimately, independent validation will be critical in revisiting the use of composite immune scores in CRC, in light of the inevitable information loss relative to their component parameters, and the need to enrich these scores with additional data points reflecting our evolving understanding of the immune contexture of solid tumors.

## Conclusion

In summary, CD3^+^ and CD8^+^ cell density counts within CRC carry overlapping but distinct prognostic significance. We identified absolute cutoffs for CD3^+^ and CD8^+^ that may define biologically meaningful prognostic subsets within CRC, and also showed that markers of intra-tumoral variation in T cell density are associated with poor prognosis. CD8+ cell density alone was as informative as combined scores in our series, and the relative density of CD3^+^ and CD8^+^ cells was the only stage-independent outcome predictor. Ongoing efforts to define comprehensive biomarkers of the immune contexture are expected to accelerate the use and benefit of emerging immunotherapy options for patients with CRC.

## Data Availability

The original contributions presented in the study are included in the article/**Supplementary Material**. Further inquiries can be directed to the corresponding author.
