# Supplementary material for: Intra-tumoral lymphocyte scoring in colorectal cancer: improving prognostic utility and correlation with underlying cancer biology
Source: Front Gastroenterol (Lausanne). 2024 Nov 28;3:1493949. doi: 10.3389/fgstr.2024.1493949 (PMC12952342; doi:10.3389/fgstr.2024.1493949)
Supplement: Supplementary file 1 [file DataSheet1.docx]

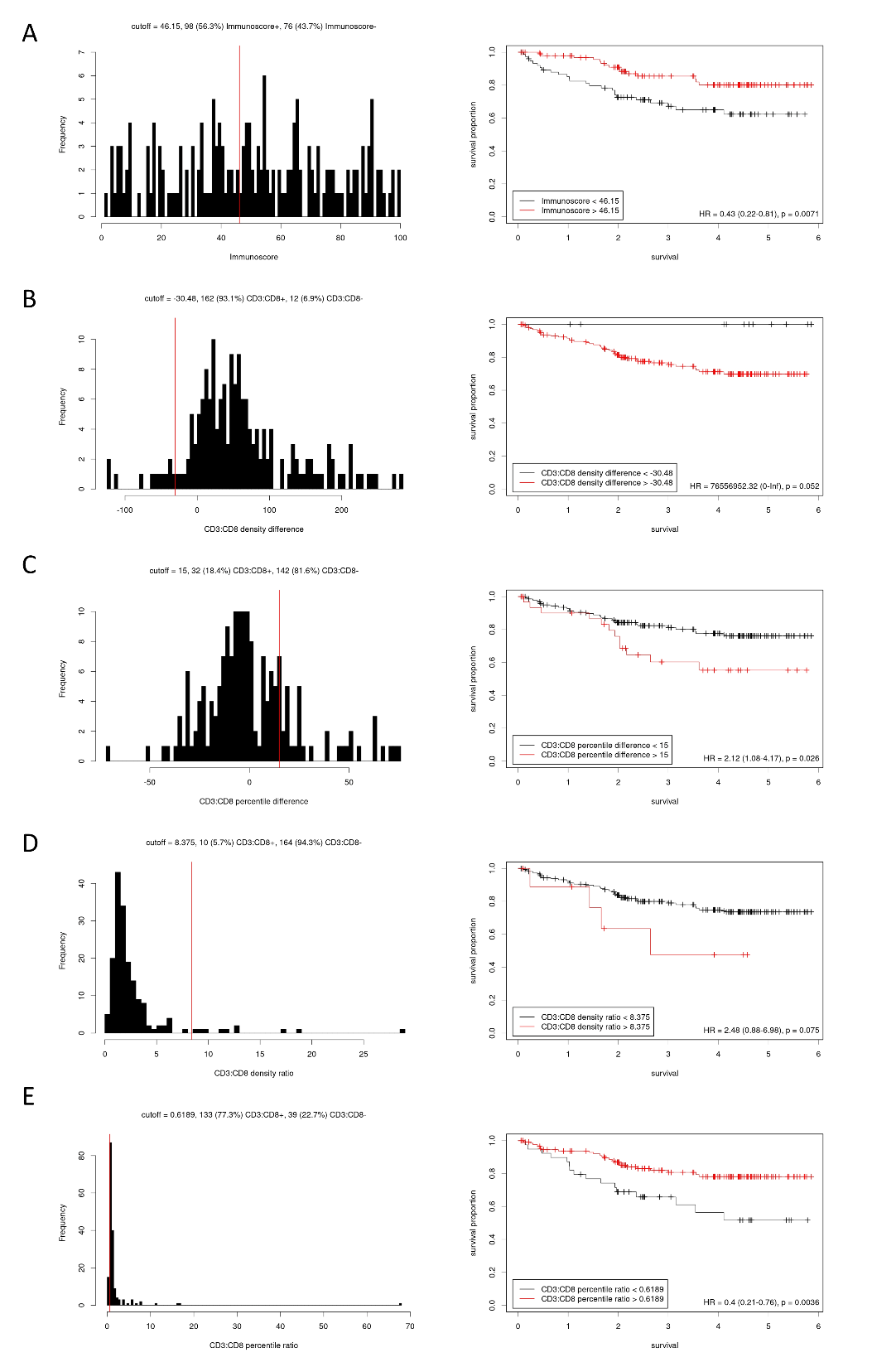


**Supplementary Figure 1**. **Histograms of exploratory combination metrics incorporating CD3+ and CD8+ cell density within colorectal cancers.** Panel A, I-score (see methods). Panel B, difference between raw CD3+ and CD8+ cell density counts. Panel C, difference between percentile-converted CD3+ and CD8+ cell density counts. Panel D, ratio between raw CD3+ and CD8+ cell density counts. Panel E, ratio between percentile-converted CD3+ and CD8+ cell density counts. Kaplan-Meier curves (disease-specific survival) for each metric were generated after stratifying cases at the indicated optimal cutpoints (Cutoff Finder, https://molpathoheidelberg.shinyapps.io/CutoffFinder_v1/; see methods).


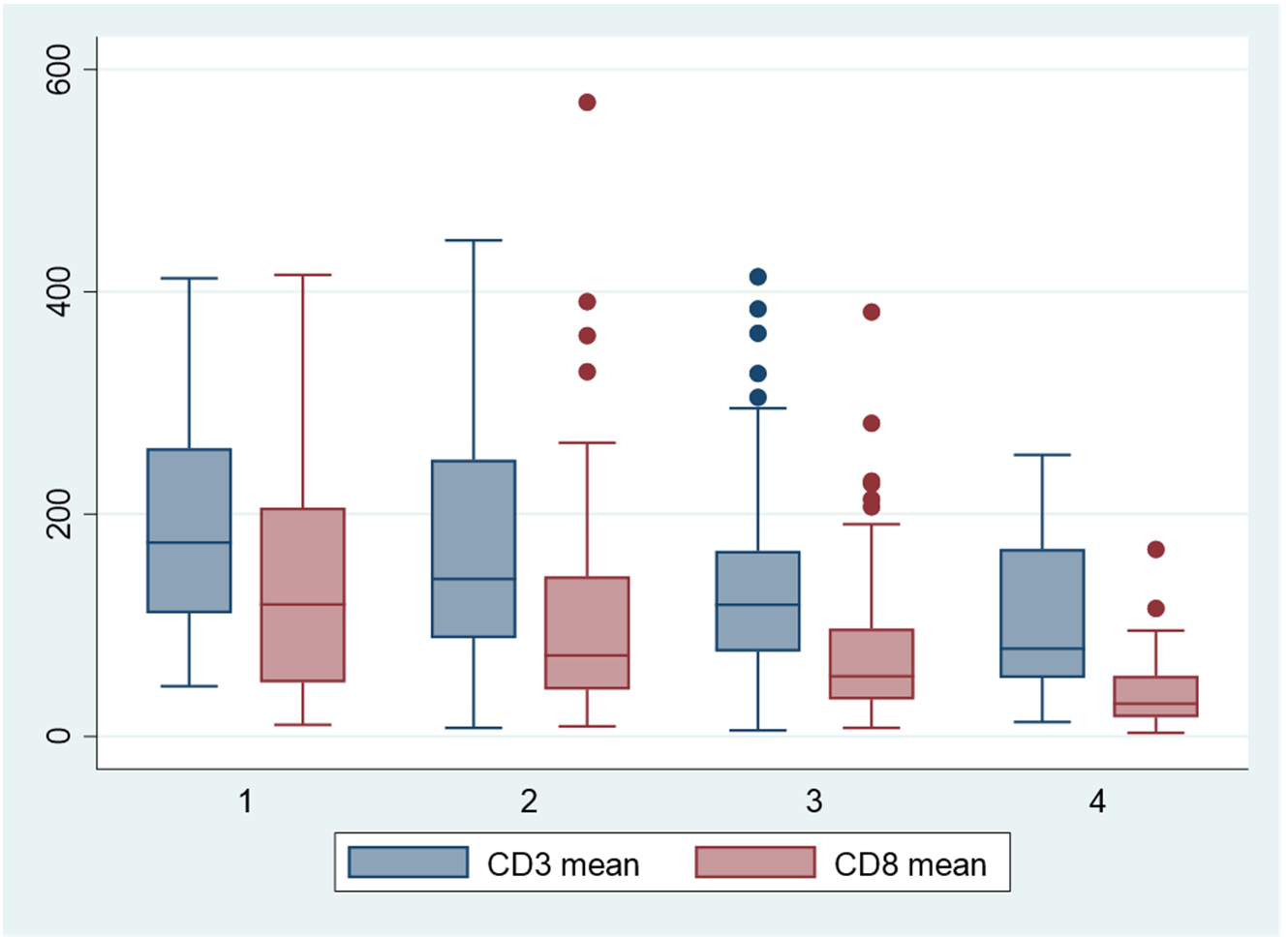


**Supplementary Figure 2**. Box plot of CD3+ and CD8+ cell density within colorectal cancers, by AJCC stage.

|  | Included  (n=201) | Unincluded  (n=100) | p |
| --- | --- | --- | --- |
| Age | 68 [60, 76] | 66 [58, 78] | 0.7 |
| Early onset CRC (age <50 years) | 19 (9.5%) | 8 (8%) | 0.8 |
| Male sex | 107 (53.2%) | 53 (53.0%) | 1 |
| Non-white race | 7 (3.5%) | 5 (5%) | 0.5 |
| Family CRC history (first-degree relative) | 23 (11.4%) | 12 (12%) | 1 |
| Type 2 diabetes | 51 (25.4%) | 18 (18%) | 0.2 |
| Body mass index | 29.4 [25.0, 33.3] | 29.4 [26.2, 33.9] | 0.4 |
| Current smoking | 23 (11.4%) | 13 (13%) | 0.7 |
| Current alcohol use | 43 (21.5%) | 32 (32%) | 0.07 |
|  |  |  |  |
| Tumor size (cm) | 4.9 [4.6, 5.3] | 4.9 [4.6, 5.3] | 0.9 |
|  |  |  |  |
| Tumor site |  |  |  |
| Right colon | 86 (42.3%) | 72 (28%) |  |
| Left colon | 115 (57.2%) | 72 (72%) | 0.02 |
|  |  |  |  |
| CEA | 3.3 [1.7, 6.9] | 3.5 [2.1, 7.2] | 0.1 |
|  |  |  |  |
| Tumor stage: |  |  |  |
| pT1 | 8 (4%) | 14 (15.7%) |  |
| pT2 | 31 (15.6%) | 11 (12.36%) |  |
| pT3 | 117 (58.8%) | 46 (51.7%) |  |
| pT4 | 43 (21.6%) | 18 (20.2%) | 0.01 |
|  |  |  |  |
| pN0 | 99 (49.5%) | 50 (56.2%) |  |
| pN1 | 69 (34.5%) | 21 (23.6%) |  |
| pN2 | 32 (16%) | 18 (20.2%) | 0.02 |
|  |  |  |  |
| pM0 | 171 (85.1%) | 80 (83.3%) |  |
| pM1 | 30 (14.9%) | 16 (16.7%) | 0.7 |
|  |  |  |  |
| I | 32 (15.9%) | 22 (22%) |  |
| II | 66 (32.8%) | 26 (26%) |  |
| III | 75 (37.3%) | 32 (32%) |  |
| IV | 28 (13.9%) | 20 (20%) | 0.2 |
|  |  |  |  |
| Overall survival hazard ratio | 1.25 [0.9, 1.74] | Ref | 0.2 |
| **Supplementary Table 1. Clinicopathologic features of included versus unincluded patients, with differences in tumor site and AJCC stage.** CRC, colorectal cancer; BMI, body mass index; CEA, carcinoembryonic antigen. Summary statistics for continuous variables are shown as median and 95% confidence interval; and for categorical variables as counts and percentages. p values shown for comparison between included vs. unincluded patients using Kruskal-Wallis test (continuous variables) and Fisher's exact test (categorical variables). | | | |

**Supplementary Table 2. Association of disease-specific survival with clinical and pathologic variables and mutation status.** CRC, colorectal cancer. MMR, mismatch repair. HR, hazard ratio. CI, confidence interval.

|  |  | HR | 95%CI | p |
| --- | --- | --- | --- | --- |
| Clinical variables | Age at diagnosis | 0.99 | [0.97, 1.01] | 0.3 |
|  | Early-onset CRC | 1.31 | [0.55, 3.09] | 0.5 |
|  | Sex | 1.82 | [1.00, 3.31] | 0.05 |
|  | Active smoking | 2.29 | [1.11, 4.75] | 0.03 |
|  | Pack-years | 1.00 | [0.98, 1.02] | 1.0 |
|  | Alcohol use | 0.39 | [0.15, 0.98] | 0.05 |
|  | Body mass index | 1.00 | [0.96, 1.04] | 0.9 |
|  | Right-sided tumor | 1.46 | [0.80, 2.66] | 0.2 |
|  | First-degree relative with CRC | 1.33 | [0.59, 3.01] | 0.5 |
|  | Chronic kidney disease | 0.62 | [0.22, 1.73] | 0.4 |
|  | Type 2 diabetes | 0.74 | [0.36, 1.53] | 0.4 |
|  | Carcinoembryonic antigen level | 1.002 | [1.001, 1.003] | <0.001 |
|  |  |  |  |  |
| Pathologic variables | pT | 3.41 | [2.06, 5.64] | <0.001 |
|  | pN | 2.47 | [1.70, 3.60] | <0.001 |
|  | pM | 6.85 | [3.80, 12.32] | <0.001 |
|  | Overall TNM stage | 3.68 | [2.46, 5.49] | <0.001 |
|  | Tumor size | 1.26 | [1.13, 1.41] | <0.001 |
|  | Tumor grade | 3.55 | [1.97, 6.42] | <0.001 |
|  | Lymphovascular invasion | 3.24 | [1.66, 6.30] | <0.001 |
|  |  |  |  |  |
|  | BRAF | 0.92 | [0.47, 1.82] | 0.8 |
|  | KRAS | 1.23 | [0.49, 3.12] | 0.7 |
|  | TP53 | 1.04 | [0.53, 2.04] | 0.9 |
|  | Any MMR gene | 0.77 | [0.38, 1.56] | 0.5 |
|  | Tumor Mutation Burden | 1.00 | [1.00, 1.01] | 0.6 |
|  |  |  |  |  |
